# Using planned behavior theory to understand cervical cancer screening intentions in Chinese women

**DOI:** 10.3389/fpubh.2023.1063694

**Published:** 2023-02-27

**Authors:** Tingting Xin, Yuting Jiang, Chunting Li, Xian Ding, Zhu Zhu, Xiao Chen

**Affiliations:** ^1^School of Medicine, Jiangnan University, Wuxi, China; ^2^Department of Anesthesiology, Affiliated Hospital of Jiangnan University, Wuxi, China; ^3^Department of Cardiology, Wuxi Second People's Hospital, Wuxi, China

**Keywords:** planned behavior theory, cervical cancer screening, intention, women, behavior

## Abstract

**Introduction:**

Cervical cancer is still one of the cancers threatening the health of Chinese women with high morbidity and mortality. However, the participation rate of cervical cancer screening (CCS) among women is low due to various reasons, so it is crucial to understand the factors that influence women's willingness to be screened for cervical cancer. This study's goal was to understand the intention of cervical cancer screening in Chinese women using the theory of planned behavior (TPB).

**Methods:**

An online questionnaire was administered to 286 women using a cross-sectional design. The questionnaire was created using the theory of planned behavior and included demographic characteristics as well as the basic structure of TPB.

**Results:**

Descriptive, correlation, and multiple linear regression models were performed to identify factors associated with cervical cancer screening behavior. 286 respondents completed the survey (95.3%). The mean scores for behavioral attitude, subjective norm and perceived behavioral control (PBC) were 32.50 (SD = 3.30), 22.59 (SD = 2.80) and 29.57 (SD = 3.37). From the regression analysis, behavioral attitude (B = 0.110, *p* = 0.001), subjective norm (B = 0.234, *p* = 0.000) and perceived behavioral control (B = 0.171, *p* = 0.000) were statistically significant in terms of intention.

**Discussion:**

This study provided a reference for improving the intention of cervical cancer screening in women.

## 1. Introduction

Cervical cancer is one of the most common cancers with a high mortality rate (1). In 2020, cervical cancer caused approximately 604,127 new cases and 341,831 deaths (2). The majority of new cervical cancer cases occurred in developing countries (3). As the world's largest developing country and a country with a large population, China has the largest burden of cervical cancer (4). According to a study conducted by Mei et al. (5), there were about 110,000 new cases of cervical cancer and about 60,000 deaths each year in China, which seriously threatened women's health. In recent years, cervical cancer survival rates have improved as a result of widespread screening and HPV vaccination (6, 7). However, due to China's vast territory, uneven economic development, insufficient medical resources, and lack of sufficient public awareness (8), cervical cancer screening (CCS) is very difficult.

showed that only one in five adult women in China said they had ever been screened (9). Past studies found that women's age, marital status, education level, income level, and medical insurance (10–13) all affected CCS rates. Many women were not fully aware of the dangers of cervical cancer to themselves and their families and believed that the risk of cervical cancer was very low without any obvious symptoms and signs, so they didn't need to attend CCS. A lack of information and awareness on CCS (14, 15) was an important reason why women did not participate in screening.

**Figure 1:**
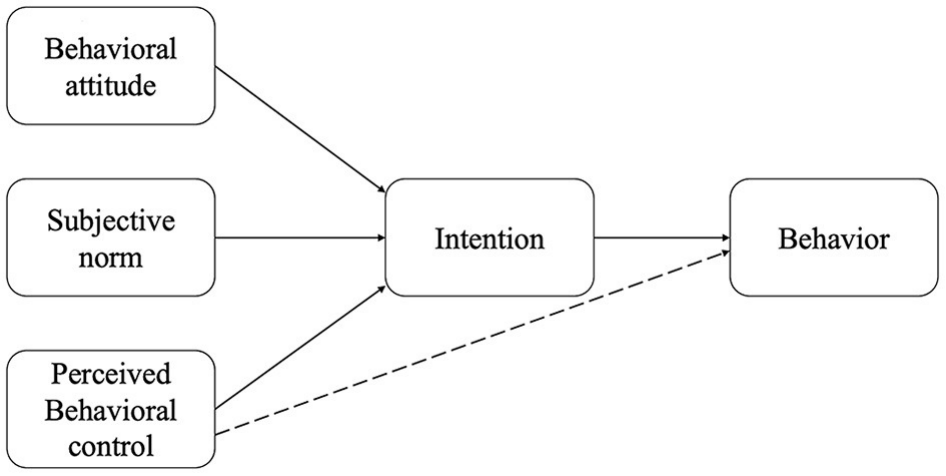
The theory of planned behavior (TPB) model.

The theory of Planned Behavior (16) (TPB) is a theory that predicts and understands particular behaviors in a particular context (Figure 1). Its essence is a social cognitive theory of decision-making processes, considered an effective theoretical framework for behavioral guidance. This theory (17) holds that behavioral intention determines behavior, and behavioral intention is controlled by behavioral attitude, subjective norm, and perceived behavior. At the same time, perceived behavioral control (PBC) can also directly affect behavior. In general, the more positive a person's attitude toward a certain behavior, the more positive the subjective norm, the more positive the PBC, and the stronger her personal behavior awareness.

A large number of studies (18–20) proved that TPB can successfully predict health behaviors, and found that TPB can effectively predict CCS (21–23). There was limited evidence of women's intentions and their predictors of CCS. Therefore, the purpose of this study was to investigate the psychological factors related to CCS intentions among Chinese women and to understand women's CCS intentions and motivations which could help to improve the CCS rate. We hypothesized the applicability of planned behavior theory (TPB) in explaining the role of Chinese women in promoting cervical cancer screening.

## 2. Materials and methods

### 2.1. Research design

This study conducted a cross-sectional study among communities in Wuxi City from March 1st to 30th, 2022. Affected by COVID-19, the questionnaire was distributed online.

### 2.2. Participants

#### 2.2.1. Inclusion criteria

1. Female aged 21–59; 2. Conscious and able to communicate effectively; 3. Volunteer and commit to completing the questionnaire.

#### 2.2.2. Exclusion criteria

1. Women diagnosed with cervical cancer; 2. Unable to communicate effectively and complete the questionnaire.

### 2.3. Sampling techniques and sample size estimation

The sampling method adopted convenience sampling and calculated the sample quantity according to the international scale formulation principle (24). One item in the scale corresponded to 5–10 samples. This scale contained 23 items and four variables, Considering the inefficiency of 10%-20% samples, and 10%-20% of sample inefficiency was considered. The sample size was calculated as follows:

Entries corresponded to sample size: (23 + 4) × 5 = 135 (23 + 4) × 10 = 270

Invalid quantity: 135 × 0.1 = 13.5 135 × 0.2 = 27 270 × 0.1 = 27 270 × 0.2 = 54

Sample size ranged: (135 + 13.5) = 148.5 (135 + 27) = 162 (270 + 13.5) = 283.5 (270 + 27) = 297

Therefore, the sample size ranged from 149 to 297, and the sample size finally selected for this survey was 300.

### 2.4. Data collection

Due to the impact of COVID-19, participants were recruited online in Wuxi City. Introduction and guidance were provided to each participant before filling in the questionnaire, The questionnaire was anonymous and collected in the online software “Questionnaire Star.”

To ascertain the intentions of Chinese women about CCS, this questionnaire was developed based on a literature review and expert panel recommendations. And semi-structured interviews were conducted with 20 female participants recruited from health facilities that were not part of the actual study. To investigate the study population's salient beliefs about the expected outcomes of utilizing CCS services, the perceived impact of social norms, and personal autonomy to seek services. Participants were invited to present their views on the benefits of using CCS services, who they believed would promote or dissuade them to use CCS services, and what hindered or facilitated their use of CCS services. In addition, the cultural relevance and linguistic intelligibility of items in the structured questionnaire were explored through pre-tests conducted before data collection.

The questionnaire was divided into two sections. The first part was the characteristics of the participants, including age, place of residence, marital status, occupation, monthly income, education level, medical insurance, and family history of cancer and cervical disease. The second part included four dimensions of the TPB structure to measure CCS willingness. Answers ranged from “strongly agree” to “strongly disagree.”

### 2.5. Ethical considerations

Jiangnan University's Medical Ethics Committee approved this study (No: JNU20220901IRB03). All investigators provided oral consent. The Ethics Committee of the Jiangnan University School of Medicine approved and waived the need for a written informed consent signature, because this study was in the form of a questionnaire survey, the risk to the subjects was less than the minimum risk, and the exemption of written signature of informed consent would not adversely affect the rights and health of the subjects. All methods were carried out in accordance with relevant guidelines and regulations.

### 2.6. Pilot study

A pilot study was conducted with 20 participants. The layout of the questionnaire was improved based on participant comments.

In “Behavior attitude,” “I feel that having regular cervical cancer screenings gives me peace of mind about my health” was changed to “I believe regular cervical cancer screening can provide me with peace of mind about my health.”

In addition, “I think cervical cancer screening can help reduce the death rate of women” was modified to “I think cervical cancer screening can help reduce mortality.”

In “Subjective norm,” “I am willing to listen to the advice of people around me to get cervical cancer screening” was adjusted to “I would like to take advice from someone close to me for cervical cancer screening.”

In “Perceived behavioral control,” “If cancer is found, I choose not to know” was changed to “I choose not to know if cancer is detected.”

In “Behavioral intention,” “After knowing the relevant information, I am willing to undergo cervical cancer screening” was modified to “I'm willing to have a cervical cancer screening now that I have learned the necessary details.”

### 2.7. Statistical analysis

All data analyses were conducted using Statistical Package for Social Science version 26 (SPSS 26.0) (25). Descriptive statistics were used to describe demographic and components of the planned behavior model of the sample. Univariate analysis was performed by calculating relative frequencies, means, and standard deviations.

As a bivariate analysis, the relationship between the theoretical constructs of intention and planned behavior was examined using Pearson's correlation analysis. Multiple linear regression models were applied to identify independent factors related to the intention to use CCS services. P < 0.05 was considered statistically significant.

Reliability referred to the stability and consistency of the results measured by the questionnaire. Internal consistency of the subscales and the entire instrument was assessed by Cronbach's alpha coefficient and split-half reliability. Generally, Cronbach's alpha coefficient of 0.70 or above was considered acceptable, and 0.80 or more was recommended.

## 3. Results

### 3.1. Sociodemographic characteristics

A total of 300 participants responded to the questionnaire, excluding low-quality questionnaires with repeated responses, and the final sample included 286 participants. The mean age was 31.62 (±8.27) years, most people (85.7%) lived in cities, and 66.4% were married. Of the participants, 8% were workers, 16.4% were technicians, 7.7% were service workers, and the rest accounted for 67.8%. 127 people (44.4%) had a monthly income of < 3,000-yuan, 3,000–5,000 yuan accounted for 24.1%, and 112 people (39.1%) had a monthly income of more than 5,000 yuan. More than half of the participants (82.2%) had an associate degree, 86.7% had health insurance, the majority (86.7%) had no family history of cancer, and 95.8% had no history of cervical disease. Table 1 displayed demographic information about the participants.

**Table 1:** Sociodemographic characteristics of participants in Wuxi, China 2022 (*n* = 286).

**Variable**	**Category**	**Frequency (*n*)**	**Relative frequency (%)**
Age	20–29years old	118	41.3
	30–39years old	127	44.4
	40–49years old	26	9.1
	≥50 years old	15	5.2
Residence	City	245	85.7
	Countryside	41	14.3
Marital Status	Single	91	31.8
	Married	190	66.4
	Separate/widowed	5	1.7
Occupation	Worker	23	8.0
	Technicist	47	16.4
	Service staff	22	7.7
	Other^a^	194	67.8
Monthly income	≤ 1,000	57	19.9
	1,000–3,000	62	21.7
	3,000–5,000	64	22.4
	≥5,000	104	36.4
Education	Middle school and below	24	8.4
	High school	27	9.4
	Associate, Bachelor's, or above	235	82.2
Medical insurance	Have	248	86.7
	Do not have	38	13.3
Family history of cancer	Have	38	13.3
	Do not have	248	86.7
History of cervical disease	Have	12	4.2
	Do not have	274	95.8

^a^Other: student, teacher, farmer, medical personnel, and so on.

### 3.2. Measurement of TPB structure

The construction of the TPB was assessed through four items and was assessed using the Likert five-scale scale. According to the measurement results, in terms of behavior attitude, 72.7% of the respondents said that it was crucial to learn about CCS, 56.3% of the respondents said that they would regret if they missed or failed to engage in CCS, and 68.9% of respondents believed that CCS for all eligible women should be conducted. In terms of subjective norms, 61.2% of the respondents said that they were more willing to get screened for cervical cancer on the advice of someone close to them, and 65.7% of the respondents believed that it was important to follow the doctor's advice to do CCS. In terms of PBC, 50.3% of the respondents believed that CCS was entirely up to them, and 55.2% of the respondents indicated that they would do CCS even if they were healthy. In terms of behavioral intentions, 53.8% of the respondents indicated that they would have regular CCS. The statistical table of cervical cancer screening based on TPB in Chinese women's intentions was shown in Table 2.

**Table 2:** Cervical cancer screening based on TPB in Chinese women's intentions (*n* = 286).

**Behavior attitude**	**Strongly agree**	**Agree**	**Neutral**	**Disagree**	**Strongly disagree**
I think it's crucial to get information about cervical cancer screening.	208 (72.7)	73 (25.5)	5 (1.7)	0	0
I believe regular cervical cancer screening can provide me with peace of mind about my health.	211 (73.8)	70 (24.5)	5 (1.7)	0	0
I think cervical cancer screening can help reduce the incidence.	203 (71.0)	70 (24.5)	10 (3.5)	3 (1.0)	0
I think cervical cancer screening can help reduce mortality.	199 (69.6)	80 (28.0)	4 (1.4)	3 (1.0)	0
Through cervical cancer screening, in my opinion, cervical disease can be identified early.	213 (74.5)	68 (23.8)	3 (1.0)	2 (0.7)	0
I will regret if I miss or fail to engage in a cervical cancer screening program.	161 (56.3)	81 (28.3)	40 (13.4)	4 (1.4)	0
I think all eligible women should have regular cervical cancer screening.	197 (68.9)	76 (26.6)	13 (4.5)	0	0
**Subjective norm**	Strongly agree	Agree	Neutral	Disagree	Strongly disagree
If I have a cervical cancer screening, my boyfriend/ husband will be supportive.	185 (64.7)	79 (27.6)	22 (7.7)	0	0
My family believe I should be screened for cervical cancer.	161 (56.3)	91 (31.8)	33 (11.5)	1 (0.3)	0
My friends encourage me to get screened for cervical cancer.	166 (58.0)	86 (30.1)	32 (11.2)	2 (0.7)	0
I would like to take advice from someone close to me for cervical cancer screening.	175 (61.2)	89 (31.1)	20 (7.0)	1 (0.3)	1 (0.3)
I believe that the guidance from my doctor is crucial for my cervical cancer screening.	188 (65.7)	86 (30.1)	8 (2.8)	4 (1.4)	0
I am willing to accept the arrangement of community/company for cervical cancer screening.	180 (62.9)	89 (31.1)	14 (4.9)	3 (1.0)	0
**Perceived behavioral control**	Strongly agree	Agree	Neutral	Disagree	Strongly disagree
I believe the decision to have cervical cancer screening is entirely up to me.	144 (50.3)	97 (33.9)	37 (12.9)	6 (2.1)	2 (0.3)
I will be embarrassed to get screened for cervical cancer.	29 (10.1)	45 (15.7)	59 (20.6)	101 (35.3)	52 (18.2)
I'd prefer to be screened by a woman doctor.	162 (56.6)	101 (35.3)	20 (7.0)	2 (0.7)	1 (0.3)
I'm going to get checked for cervical cancer even if I'm healthy.	158 (55.2)	91 (31.8)	33 (11.5)	4 (1.4)	0
I will still have the cervical cancer screening even though it may be uncomfortable.	134 (46.9)	105 (36.7)	41 (14.3)	5 (1.7)	1 (0.3)
I will participate in cervical cancer screening if it can be more convenient.	193 (67.5)	81 (28.3)	9 (3.1)	2 (0.7)	1 (0.3)
I choose not to know if cancer is detected.	15 (5.2)	11 (3.8)	35 (12.2)	89 (31.1)	136 (47.6)
**Behavioral intention**	Strongly agree	Agree	Neutral	Disagree	Strongly disagree
I'm willing to have a cervical cancer screening now that I have learned the necessary details.	171 (59.8)	93 (32.5)	20 (7.0)	1 (0.3)	1 (0.3)
If possible, in the future, I would be open to getting screened for cervical cancer.	181 (63.3)	94 (32.9)	7 (2.4)	2 (0.7)	2 (0.7)
I will definitely have regular cervical cancer screening.	154 (53.8)	90 (31.5)	41 (14.3)	1 (0.3)	0

### 3.3. Average score for TPB structures

The mean score of TPB structures was calculated using descriptive statistics. The mean scores for direct attitude, subjective norm, and PBC were 32.5 (SD = 3.3), 22.6 (SD = 2.8), and 29.6 (SD = 3.4), respectively. The mean score for the intention was 13.5 (SD = 1.8). The descriptive statistical table of the components of the planned behavior model of Chinese women was shown in Table 3.

**Table 3:** Descriptive statistics of the components of the Chinese women's planned behavior model.

**Constructs**	**Number of items**	**Mean**	**SD**
Behavior attitude	7	32.5	3.3
Subjective norm	6	22.6	2.8
Perceived behavioral control	7	29.6	3.4
Behavioral intention	3	13.5	1.8
Total	23	-	-

PBC, direct perceived behavioral control; SD, Standard deviation, α-value.

### 3.4. Correlation analysis results

#### 3.4.1. Relationships between intention, sociodemographic factors, and measures of TPB structure

All necessary bivariate analyses were performed to explore associations between dependent and independent variables. Sociodemographic variables included age, place of residence, marital status, occupation, monthly income, educational level, medical insurance, family history of cancer, and history of cervical disease. The findings demonstrated that there was no discernible relationship between occupation and educational attainment and CCS intention. Variables that became important factors were age, place of residence, marital status, income, medical insurance, family history of cancer, and history of cervical disease.

The link between the elements of the TPB structural model and women's readiness to screen for cervical cancer was investigated using a Pearson correlation analysis. The results of the study showed that the various structures of the TPB structure were positively correlated with the CCS intention. Table 4 showed the correlation between TPB model and intention.

**Table 4:** Correlation between Pearson's TPB model and female intentions (*n* = 286).

**Component**	**Behavioral intention**	**Behavior attitude**	**Subjective norm**	**Perceived behavioral control**
Behavioral intention	1			
Behavior attitude	0.714^**^	1		
Subjective norm	0.761^**^	0.816^**^	1	
Perceived behavioral control	0.714^**^	0.655^**^	0.708^**^	1

^**^the correlation is significant at P < 0.01.

### 3.5. Regression and collinearity analysis results

To predict behavior intentions for CCS use, a simple linear regression analysis was conducted using all TPB constructs and variables that were significant in the bivariate analysis. Attitudes, subjective norms, and PBC of CCS were included in a multivariate linear analysis. Standardized regression coefficient subjective norm was found to be the best factor (B = 0.234, *p* < 0.01) followed by PBC (B = 0.171, *p* < 0.01). These showed that women who believed significant others would support their use of CCS services were 23.4% more likely to use CCS than their peers, holding other things constant. A 17.1% increase in positive unit-changed intentions for perceived control of environmental facilitators' beliefs about using CCS services. Similarly, a unit-positive shift in women's perceptions of the benefits of using CCS services would boost someone's intention to utilize them by 11% if all other variables remained constant. In this study, it was discovered that attitude was the least component connected with the intention to utilize CCS. (B = 0.110, *p* < 0.01). Table 5 showed the independent factors related to the behavioral intention of cervical cancer screening.

**Table 5:** Independent factors associated with behavioral intentions of cervical cancer screening in Wuxi, China 2022.

**Model**	**Unstandardized coefficients**		**Standardized coefficients**	**t**	**Sig**.	**Multicollinearity statics**	
	**B**	**Std. Error**	**Beta**		**Tolerance**	**VIF**	
(Constant)	−0.448	0.655		−0.685	0.494		
Attitude	0.110	0.033	0.204	3.299	0.001	0.322	3.107
Subjective norm	0.234	0.042	0.367	5.550	0.000	0.281	3.558
PBC	0.171	0.027	0.321	6.364	0.000	0.481	2.077

### 3.6. Reliability

Cronbach's alpha reliability and split-half reliability of the whole questionnaire were 0.912 and 0.894, and the coefficients of each subscale ranged from 0.903 to 0.931, showing a certain internal consistency.

## 4. Discussion

The study found that more than half of the respondents (53.8%) said they would have regular CCS. Variables that became important factors were age, place of residence, marital status, income, medical insurance, family history of cancer, and history of cervical disease. Knowing and accessing the information on cervical cancer prevention was well known to be critical, and lack of awareness was shown to be one of the major barriers to CCS (26). The majority of respondents (72.7%) said it was critical to obtain information on CCS. Previous research suggested that knowledge may positively influence attitudes and thus affect individuals' intentions to screen. Health professionals were the most common source of information, and the survey found that 65.7% of respondents in this study said that doctor's advice was important, suggesting the importance of doctor-patient communication in improving screening compliance. A study indicated that health education efforts for Unmarried women should be targeted (27).

As mentioned by Ajzen, the intention to engage in behavior was influenced by the attitude, subjective norm, and PBC and varied based on the behavior, the population being studied, the setting, and the time of the investigation. This study showed that women's willingness to use CCS was primarily influenced by subjective norms, followed by PBC of CCS, with attitude identified as the least important predictive construct of planned behavior theory. However, one study found that (28) PBC was the strongest predictor of intention, followed by subjective norm to be screened for cervical cancer. This may be due to differences in the context of the studies.

Subjective norm was the strongest intention-related factor, consistent with a previous study (29). This meant that women who were more strongly supportive of their significant other wanting them to be screened for cervical cancer expressed a stronger willingness. According to the data (30), family support, particularly from female relatives, was an important facilitator of screening and treatment. According to the report, women prioritized their family's health over their own, and some women had fatalistic beliefs about cancer. Younger women often sought advice from older female relatives, so efforts should be made to focus on women of all ages. 64.7% of respondents indicated that partner support was important, so it was important for interveners to ensure that women felt supported by significant others and increase their confidence to overcome difficulties, thereby improving the intention to be screened for cervical cancer.

In this study, the least important and positively correlated factor with the willingness to undergo CCS was attitude. This was consistent with previously conducted research (31). This study provided support for a theory-based cancer screening program. This study well-applied concepts from the theory of planned behavior to identify predictors of behavioral intentions in women with cervical cancer. Cervical cancer is a preventable and controllable disease, and effectively targeted programs must be developed to encourage women to undergo CCS, thereby reducing cervical cancer-related health problems.

## 5. Limitations

This study did not consider other variables such as cervical cancer knowledge and screening results for cervical cancer in the past as indicators of future behavior, because there was no national screening program for urban areas, the survey results in Wuxi may not be representative of screening for the national female population. This study evaluated and analyzed the influencing factors of female cervical cancer screening in Wuxi from four aspects of the theory of planned behavior, which can provide a reference for the future development of planned and targeted measures to improve the screening rate.

## 6. Conclusions

From this study, it can be seen that the predictors of female CCS behavioral intention are attitude, subjective norm, and PBC. Therefore, strategies and actions should be needed to improve women's attitudes and sense of control toward CCS among women and the influencers around them, thereby increasing women's willingness to be screened for cervical cancer.

## Data availability statement

The original contributions presented in the study are included in the article/supplementary material, further inquiries can be directed to the corresponding author.

## Ethics statement

The studies involving human participants were reviewed and approved by the Medical Ethics Committee of Jiangnan University. Written informed consent for participation was not required for this study in accordance with the national legislation and the institutional requirements.

## Author contributions

XD and TX designed the study. YJ provided resources and analysis methods. CL, ZZ, and YJ collected data. TX analyzed data and wrote the original manuscript. XC revised manuscript. All authors have read and agreed to the published version of the manuscript.

## References

[1] Arbyn M Weiderpass E Bruni L de Sanjosé S Saraiya M Ferlay J. Estimates of incidence and mortality of cervical cancer in 2018: a worldwide analysis. Lancet Glob Health. (2020) 8:e191–203. 10.1016/S2214-109X(19)30482-6 31812369 PMC7025157

[2] Sung H Ferlay J Siegel RL Laversanne M Soerjomataram I Jemal A. Global cancer statistics 2020: GLOBOCAN estimates of incidence and mortality worldwide for 36 cancers in 185 countries. CA Cancer J Clin. (2021) 71:209–49. 10.3322/caac.21660 33538338

[3] Ferlay J Soerjomataram I Dikshit R Eser S Mathers C Rebelo M. Cancer incidence and mortality worldwide: Sources, methods and major patterns in GLOBOCAN 2012. Int J Cancer. (2015) 136:E359–86. 10.1002/ijc.29210 25220842

[4] Di J Rutherford S Chu C. Review of the cervical cancer burden and population-based cervical cancer screening in China. Asian Pac J Cancer Prev. (2015) 16:7401–7. 10.7314/apjcp.2015.16.17.7401 26625735

[5] Mei Z Heling B Limin W Zhenping Z Zhengjing H Xiao Z. Analysis of cervical cancer screening and related factors in China. Natl Med J China. (2021) 101:1869–74 10.3760/cma.j.cn112137-20210108-00054 34192843

[6] Sasieni P Castanon A Cuzick J. Effectiveness of cervical screening with age: population based case-control study of prospectively recorded data. BMJ. (2009) 339:b2968. 10.1136/bmj.b2968 19638651 PMC2718082

[7] Lei J Ploner A Elfström KM Wang J Roth A Fang F. HPV vaccination and the risk of invasive Cervical Cancer. N Engl J Med. (2020) 383:1340–8. 10.1056/NEJMoa1917338 32997908

[8] Wang B He M Chao A Engelgau MM Saraiya M Wang L. Cervical cancer screening among adult women in China, 2010. Oncologist. (2015) 20:627–34. 10.1634/theoncologist.2014-0303 25956407 PMC4571778

[9] Lin W Chen B Wu B Yuan S Zhong C Huang W. Cervical cancer screening rate and willingness among female migrants in shenzhen, china: three-year changes in citywide surveys. Cancer Res Treat. (2021) 53:212–22. 10.4143/crt.2020.219 32878425 PMC7812020

[10] Chan CWH Choi KC Wong RS Chow KM So WKW Leung DYP. Examining the cervical screening behavior of women aged 50 or above and its predicting factors: a population-based survey. Int J Environ Res Public Health. (2016) 13:E1195. 10.3390/ijerph13121195 27918456 PMC5201336

[11] Chang HK Myong JP Byun SW Lee SJ Lee YS Lee HN. Factors associated with participation in cervical cancer screening among young Koreans: a nationwide cross-sectional study. BMJ Open. (2017) 7:e013868. 10.1136/bmjopen-2016-013868 28373252 PMC5387966

[12] Heling B Linhong W Limin W Liwen F Mei Z Zhenping Z. Study on the coverage of cervical and breast cancer screening among women aged 35-69 years and related impact of socioeconomic factors in China, 2013. Chin J Epidemiol. (2018) 39:208–12. 10.3760/cma.j.issn.0254-6450.2018.02.014 29495207

[13] Bao H Zhang L Wang L Zhang M Zhao Z Fang L. Significant variations in the cervical cancer screening rate in China by individual-level and geographical measures of socioeconomic status: a multilevel model analysis of a nationally representative survey dataset. Cancer Med. (2018) 7:2089–100. 10.1002/cam4.1321 29573569 PMC5943548

[14] Liu T Li S Ratcliffe J Chen G. Assessing Knowledge and Attitudes towards Cervical Cancer Screening among Rural Women in Eastern China. Int J Environ Res Public Health. (2017) 14:967. 10.3390/ijerph14090967 28846616 PMC5615504

[15] Taneja N Chawla B Awasthi AA Shrivastav KD Jaggi VK Janardhanan R. Knowledge, attitude, and practice on cervical cancer and screening among women in india: a review. Cancer Control. (2021) 28:10732748211010800. 10.1177/10732748211010799 33926235 PMC8204637

[16] Ajzen I. The theory of planned behavior. Organ Behav Hum Decis Process. (1991) 50:179–211. 10.1016/0749-5978(91)90020-T

[17] Ajzen I. Constructing a Theory of Planned Behavior Questionnaire. (2022). Available online at: https://people.umass.edu/aizen/tpb.html (accessed Oct 4, 2022).

[18] Khani Jeihooni A Darvishi N Harsini PA. The effect of educational intervention based on the theory of planned behavior on mammography screening in Iranian women. J Cancer Educ. (2020) 35:264–73. 10.1007/s13187-018-1460-3 30604386

[19] Di Sarra L Ghezzi V Eastland TY Antonini F Scialó G Zega M. Applying the theory of planned behavior to explain women's role in prostate cancer screening. Res Theory Nurs Pract. (2015) 29:200–13. 10.1891/1541-6577.29.3.200 26502556

[20] Forbes CC Blanchard CM Mummery WK Courneya KS A. comparison of physical activity correlates across breast, prostate and colorectal cancer survivors in Nova Scotia, Canada. Support Care Cancer. (2014) 22:891–903. 10.1007/s00520-013-2045-7 24240648

[21] Roncancio AM Ward KK Fernandez ME. Understanding cervical cancer screening intentions among latinas using an expanded theory of planned behavior model. Behav Med. (2013) 39:66–72. 10.1080/08964289.2013.799452 23930898 PMC4895917

[22] Abamecha F Tena A Kiros G. Psychographic predictors of intention to use cervical cancer screening services among women attending maternal and child health services in Southern Ethiopia: the theory of planned behavior (TPB) perspective. BMC Public Health. (2019) 19:434. 10.1186/s12889-019-6745-x 31023306 PMC6482500

[23] Yang S Huang LH Zhao XH Xing MY Shao LW Zhang MY. Using the Delphi method to establish nursing-sensitive quality indicators for ICU nursing in China. Res Nurs Health. (2019) 42:48–60. 10.1002/nur.21925 30681165

[24] Watson R Thompson DR. Use of factor analysis in journal of advanced nursing: literature review. J Adv Nurs. (2006) 55:330–41. 10.1111/j.1365-2648.2006.03915.x 16866827

[25] Areklett EW Fagereng E Bruheim K Andersson S Lindemann K. Self-reported cognitive impairment in cervical cancer survivors: a cross-sectional study. Psychooncology. (2022) 31:298–305. 10.1002/pon.5818 34516040

[26] Chidyaonga-Maseko F Chirwa ML Muula AS. Underutilization of cervical cancer prevention services in low- and middle-income countries: a review of contributing factors. Pan Afr Med J. (2015) 21:231. 10.11604/pamj.2015.21.231.6350 26523173 PMC4607967

[27] Hertzum-Larsen R Kjær SK Frederiksen K Thomsen LT. Participation in cervical cancer screening among immigrants and Danish-born women in Denmark. Prev Med. (2019) 123:55–64. 10.1016/j.ypmed.2019.02.023 30796926

[28] Roncancio AM Ward KK Sanchez IA Cano MA Byrd TL Vernon SW. Using the theory of planned behavior to understand cervical cancer screening among Latinas. Health Educ Behav. (2015) 42:621–6. 10.1177/1090198115571364 25712240 PMC4932857

[29] Jalilian F Emdadi S. Factors related to regular undergoing pap-smear test: application of theory of planned behavior. J Res Health Sci. (2011) 11:103–8. 10.21203/rs.3.rs-58161/v1 22911960

[30] Madhivanan P Valderrama D Krupp K Ibanez G. Family and cultural influences on cervical cancer screening among immigrant Latinas in Miami-Dade County, USA. Cult Health Sex. (2016) 18:710–22. 10.1080/13691058.2015.1116125 26671002

[31] Bish A Sutton S Golombok S. Predicting uptake of a routine cervical smear test: a comparison of the health belief model and the theory of planned behaviour. Psychol Health. (2000) 15:35–50. 10.1080/08870440008400287

